# Special Issue “Viral Infections in Developing Countries”

**DOI:** 10.3390/v14020405

**Published:** 2022-02-16

**Authors:** Fabrício Souza Campos, Luciana Barros de Arruda, Flávio Guimaraes da Fonseca

**Affiliations:** 1 Laboratório de Bioinformática & Biotecnologia, Campus de Gurupi, Universidade Federal do Tocantins, Gurupi 77402-970, TO, Brazil; 2 Departamento de Virologia, Instituto de Microbiologia Paulo de Góes, Universidade Federal do Rio de Janeiro, Rio de Janeiro 21941-902, RJ, Brazil; 3 Laboratório de Virologia Básica e Aplicada, Departamento de Microbiologia, Instituto de Ciências Biológicas, Universidade Federal de Minas Gerais, Belo Horizonte 31270-901, MG, Brazil

Viral infections by endemic, emerging, and reemerging viruses are constantly challenging public health systems and health policies all over the world. The circulation of these viruses in developing countries is often neglected and presses for the development of new cost-effective diagnostic methods, therapeutics and prevention strategies, and efficient epidemiological surveillance. Despite particular economic and public health adversities, developing countries have been braving these issues and making important contributions to virology research over the years. Thus, this Special Issue of *Viruses* is a collection of the high-quality science developed in the face of the challenges experienced until 2021, including the rapid and severe spread of SARS-CoV2, causing the worst pandemic of the last 100 years, with 375.9 million cases and 5.7 million deaths in 224 countries to date [1].

The COVID-19 pandemic has affected developed and under-developed countries indistinctly. Nonetheless, developing nations are already pressed by economic and social problems that are further stressed in a moment such as this, giving rise to vicinal problems that may include outbreaks of other diseases, including those of viral etiology. In this Special Issue, we are proud to have received a total of 41 manuscript submissions with an acceptance rate of 60.1% (25 papers) from colleagues from developed and developing countries working on a wide range of different viruses. Importantly, most of the studies were fully performed in developing countries from South America, Africa, and Asia, but studies developed in North and Central America and Europe are also part of this collection, alongside collaborations between groups from different continents, supporting that solid scientific globalization generates relevant high-quality science.

In the face of the current pandemic, many of the published manuscripts are related to SARS-CoV-2. Paiva et al. [2] sequenced 101 strains of SARS-CoV-2 from patients presenting COVID-19 symptoms that reside in Pernambuco and showed multiple introductions followed by the ongoing community spread of SARS-CoV-2 at one of the largest metropolitan areas of Northeast Brazil. In another study, de Souza et al. [3] described mutational events across samples from Brazilian SARS-CoV-2 sequences available on Global Initiative on Sharing Avian Influenza Data-EpiCoV (GISAID-EpiCoV) [4] and estimated the number of genomes necessary to report a new variant. The authors also reported that the virus genomic diversity detected in Brazil and Chile in South America was similar to that detected in South Africa and India, and all represent potential hotspots for the generation of new variants, especially when social restrictions are not strictly applied, leading to increased viral circulation. Engelbrecht et al. [5], through the complete sequencing of 46 genomes and subsequent phylogenetic reconstruction, showed at least nine early introductions of SARS-CoV-2 in Cape Town, South Africa. Thus, the authors claim that genomic surveillance has been successfully used to investigate and track the spread of early SARS-CoV-2 introductions in Cape Town. Zeghbib et al. [6] explored the evolutionary, genetic, and epidemiological aspects regarding the Algerian SARS-CoV-2 pandemic, aptly demonstrating the multiple introductions of the disease and the heterogeneity of the genomes. Additionally, research findings revealed unique amino acid substitutions by characterizing the mutational patterns and the effect on the corresponding proteins. Cedro-Tanda et al. [7] reported the emergence and spread of the new B.1.1.519 variant in Mexico City in the beginning of 2021, and described its evolution, transmissibility, and association with relevant clinical traits. The authors observed that variant B.1.1.519 was significantly associated with severe disease, hospitalization, and death.

Zhao et al. [8] described a statistical learning strategy using generalized additive models, unsupervised learning techniques, and single-nucleotide polymorphism methodologies for identifying and spatiotemporally characterizing viral variants where a Spike protein mutation is significantly present in a given geographic area. The study provided detailed information on the emergence and persistence of specific mutations in US states, helping to identify mutations that may warrant further investigation.

Beyond surveillance, diagnostic methods and experimental models of infection were also discussed. Costa et al. [9] developed a methodology based on a reverse transcription-quantitative real-time polymerase chain reaction (RT-qPCR) to detect SARS-CoV-2 RNA in pool samples from adults attending a public emergency care unit, a reference for COVID-19 in Belo Horizonte, Brazil. Even samples on the edge of detectability in individual testing were detected correctly. A recommended increase in cut-off values relative to the RT-qPCR original cut-off was calculated in order to compensate for the dilution caused by pooling. This may enhance the contribution of pool testing to large-scale testing for COVID-19. Rosa et al. [10] summarized the main in vitro and in vivo models of SARS-CoV-2 infection developed so far and discussed their advantages, drawbacks, and main uses. The authors concluded that these models have been applied in different research areas, such as virus characterization and the development of vaccines and antiviral therapies, and the limitations of each model must be considered during experimental design.

Coronaviruses affecting domestic animals (pets or livestock) or wildlife represent a problem in the current one-health view and approaches in relation to infectious diseases with pandemic potential. The Middle East respiratory syndrome-related coronavirus (MERS-CoV) is a persistent zoonotic pathogen with frequent spillover from dromedary camels (natural reservoir) to humans in the Arabian Peninsula, resulting in limited outbreaks of MERS with a high case–fatality rate. In a recent, not-peer-reviewed article, Chinese researchers described the presence of ACE2-binding relatives of MERS-CoV in African bats, further stressing the continuous risk of coronavirus spillover to humans. In our Special Issue, Seifert et al. [11] discuss the low MERS-CoV genetic diversity detected in Jordan camels, consistent with a lack of endemic circulation in these camel herds and reflective of data from MERS outbreaks in humans. Study data suggest the transmission of MERS-CoV among two camel herds in Jordan in 2016 following a single introduction event. Thus, the authors hypothesize that camel management practices, including import and export and herd size, influence the diversity and maintenance of MERS-CoV in the natural reservoir.

Regarding further investigations on interactions between human respiratory viruses and host cells, an elegant study performed by Souza Cardoso et al. [12] assessed the replication of human respiratory syncytial virus (HRSV) in a human CD4+ T cell line. The authors demonstrated that A3.01 cells are susceptible but virtually not permissive to HRSV infection, in contrast to Hep-2 epithelial cells, in which virus-productive infection results in cell death. Multiple replication steps were hampered in CD4 T-cells, including virus fusion, the formation of inclusion bodies and possibly the traffic of viral proteins to the plasma membrane. These findings suggest that differences in virus–host cell interaction among distinct cell types may contribute to the observed cytopathic effect or long-term infection in the tissues.

Arboviruses such as Dengue virus (DENV), Chikungunya virus (CHIKV), and Yellow fever virus (YFV), also have been frequently associated with outbreaks and epidemics, causing diseases with high morbidity and mortality rates in developed and undeveloped countries, sometimes under a deficient surveillance program. Mwanyika et al. [13] presented a systematic review and meta-analysis analyzing the prevalence of Dengue infection and associated risk factors in Africa from 1960 to 2020. The authors highlight an increased risk of severe disease in Africa due to the increasing circulation of multiple DENV serotypes. Thus, there is a need for routine laboratory dengue diagnosis in Africa to facilitate the early detection of cases, provisions for appropriate patient care, the identification of serotypes/genotypes, and outbreak preparedness. Moreover, it is important to implement effective mosquito surveillance to identify hotspots, and the promotion of education on individual behaviors and environmental management practices that can limit the spread of Dengue infection in Africa. Paradkar et al. [14] explored Dengue surveillance in Mumbai, and discussed the necessity of novel and integrated approaches to improve disease detection, mosquito surveillance, and the control of mosquito-borne diseases, beyond the use of insecticides and the current activities to reduce mosquito breeding sites. Soe et al. [15] analyzed DENV-1-4 serotypes in 1235 serum samples collected in Myanmar between 2017 and 2019 and showed that most DENV-1–4 strains had been circulating for several years. The authors also reported the emergence of DENV-3 genotype-I in 2017 samples, which coincided with a period of increased DENV-3 cases and marked changes in the serotype dynamics.

Chikungunya virus is a single-stranded RNA virus of the Togaviridae family and genus Alphavirus originating from sub-Saharan Africa, where sylvatic and urban cycles are recognized. The urban cycle can be caused by Aedes albopictus, originating from Southeast Asia. In early 2019, a sudden increase in acute fever/arthralgia cases was detected in Matadi in the Democratic Republic of the Congo, and CHIKV was investigated as the causal agent. Weggheleire et al. [16] assessed the epidemiological, clinical, laboratory, and entomological characteristics of the outbreak and the perception of the population regarding the outbreak. The investigation confirmed the first major CHIKV outbreak in Matadi, with Ae. albopictus as the main driver, highlighting the necessity of the development of point-of-care diagnostics and appropriate clinical care, and moreover, vector control strategies.

Yellow fever (YF) is an acute infectious febrile disease caused by the YFV. It is a member of the family Flaviviridae and genus Flavivirus. YFV is transmitted by the bite of mosquito vectors and has two transmission cycles, a wild (rural or forest area) and an urban, with different vectors depending on the cycle and the region. Moreover, monkeys usually die (named epizooties) when affected by YF. Thus, dead animals are important sentinels that indicate viral circulation but do not transmit the disease to humans. In order to control the disease, in addition to vaccination, it is important to carry out an intense and integrated surveillance program aimed at detecting epizootic cases, capturing vector mosquitoes, and diagnosis using genetic and sequencing tools, in addition to educational work on the epidemiology and risk of disease, which can lead to the death of unvaccinated people. In that regard, Diagne et al. [17] report an outbreak that occurred in eastern Senegal between 2020 and 2021. Virological analyses highlighted the implication of sylvatic mosquito species in virus transmission. Genomic analysis showed a close relationship between the circulating strain in eastern Senegal, 2020, and another from the West African lineage previously detected in 2018 from an unvaccinated Dutch traveler who visited Gambia and Senegal before developing symptoms after returning to Europe. In another study, Andrade et al. [18] showed the role of genomic surveillance in determining the pathways of distribution of the virus and in providing references for the implementation of preventive measures for populations in high-risk areas. The authors collected samples of non-human primates in the state of Rio Grande do Sul (RS), Brazil, causing the state to declare a Public Health Emergency of State Importance, despite no human cases reported. Next, YFV near-complete genomes recovered from the outbreak were sequenced and examined, aiming at a better understanding of the virus distribution. The results suggest that the most likely sequence of events involved the reintroduction of YFV from the Southeast region to the Southern region of Brazil, causing YFV to re-emerge in the southernmost state of Brazil at the end of 2020.

Hepatitis means inflammation of the liver and can be often caused by viral infections. It may present in acute form as a recent infection with relatively rapid onset, or in chronic form. The most common types of viral hepatitis are hepatitis A, B, C, D and E. Viral hepatitis is either transmitted through contaminated food or water (A, E) or via blood and body fluids (B, C). Hepatitis A virus (HAV) affects millions of people worldwide. Moreover, most regions in Africa and Southeast Asia are highly endemic. In South Africa, hepatitis A is a notifiable medical condition, requiring notification by clinicians and by testing laboratories. Thus, Prabdial-Sing et al. [19] utilized the number of laboratory-confirmed hepatitis A cases nationally from 2017 to 2019 to calculate testing and incidence rates, and to determine thresholds for public health action. The study showed an increased incidence of hepatitis A in children 1 to 9 years of age. In addition, there is a transition to intermediate endemicity as the average age of infection shifts from children to older age groups, in which disease is more severe.

The exposure to pathogens and risk situations occur differently according to each individual, region and social group. This is also true for viral hepatitis. Thus, Nascimento et al. [20] identified the presence, genotypes and factors associated with hepatitis E virus (HEV) exposure among a community of people who use crack cocaine (PWUCC) in northern Brazil. Blood and fecal samples were collected and tested for HEV using an immunoenzymatic assay, and the genotype was identified by PCR. Logistic regressions were used to identify the risk factors independently associated with exposure to HEV. In total, 18.1% of PWUCC were exposed to HEV and HEV RNA was detected in fecal and blood samples. The subtype 3c was identified in all of the samples. The factors associated with exposure to HEV were low monthly income, unstable housing, crack cocaine use ≥40 months, and the shared use of crack cocaine equipment. The authors highlight the urgent need for improved diagnosis, prevention, and treatment intervention, and the provision of help to PWUCC. In another study, Silva et al. [21] made a multi-site cross-sectional study of people who use illicit drugs (PWUDs) in the same Amazon region, aiming at the detection and genetic characterization of hepatitis B (HBV) and D (HDV) viruses. In total, 1074 blood samples were collected from PWUDs from the Brazilian Amazon. HBV and HDV were detected by ELISA and PCR. Viral genotypes were identified by nucleotide sequencing followed by phylogenetic analysis. Markers for HBV were detected varying between serological (32.2%) and molecular (7.2%), including sub-genotypes A1, A2, D4, and F2a. Among PWUDs with HBV DNA, serological (19.5%) and molecular (11.7%) HDV markers were detected, such as HDV genotypes 1 and 3. The authors conclude with the urgent need for viral hepatitis prevention and treatment in PWUDs.

The review by Machado et al. [22] described 30 years of studies at the Virus Laboratory at the Federal University of Pará, Brazil, addressing the prevalence and molecular epidemiology of HIV-1, HTLV-1/2, HPV, HBV, Treponema pallidum, and Chlamydia trachomatis among urban and non-urban populations, and also in vulnerable groups in the Brazilian Amazon. The authors discussed the challenges and advances in detecting and preventing sexually transmitted infections considering this immense geographic region, where essential health services are unavailable to the entire population, especially the most vulnerable, such as female sex workers, illicit drug users, remnants of enslaved populations (named quilombolas) and indigenous communities.

Willim et al. [23] analyzed the prevalence of pre-antiretroviral therapy (ART) drug resistance mutations (DRMs) in a Kenyan population and observed high levels of HIV drug resistance against all classes of antiretroviral drugs, including the current first-line ART regimens in Africa. The authors also detected some correlation between DRMs and host HLA class I genes and suggested that the development of DRMs may be influenced by HLA class I-restricted immunity.

Within the retrovirus family of viruses, feline leukemia virus (FeLV) is specific to members of the cat family and does not pose a risk to other species of animals or people. The article of Cano-Ortiz et al. [24] proposes a new method for FeLV classification based on the molecular analysis of the surface envelope (SU) gene. For this, 404 publicly available SU sequences were used to reconstruct a maximum likelihood tree. However, only 63 of these sequences had available information about phenotypic tests or subgroup assignments. Thus, the authors propose that phylogenetic and recombination analysis together can explain the current phenotypic classification of FeLV viruses.

Human pathogens are also frequently transmitted through contaminated water. Rotavirus A (RVA) has been considered the main cause of diarrheal disease in children under five years in emergency services in both developed and developing countries. With this in mind, Martinez-Gutierrez et al. [25] performed a retrospective study with a previously reported detection of the G3P{8} strain collected from a child who presented with acute gastroenteritis. A near-full genome phylogenetic analysis confirmed the presence of the novel equine-like G3P{8} with a Wa-like or genogroup 1 backbone for the first time in Colombia, demonstrating the importance of the surveillance of emerging viruses in the Colombian population.

Using a different approach for virus surveillance, Abdelrahman et al. [26] evaluated the prevalence of human parvovirus (B19V), a causative agent of erythema infectiosum in children, in blood donors from different nationalities residing in Qatar. Analysis of the seroprevalence, viremia rate, and circulating genotypes of B19V showed a relatively high seroprevalence, although only 2.1% of the samples were IgM-positive and 1.4% had detectible B19V DNA. The authors suggested that blood banks in Qatar might need to consider screening for B19V, especially when transfusion is intended for high-risk populations, including immunocompromised patients.

Finally, Sousa-Junior et al. [27] presented a review paper discussing several aspects of the most prevalent viruses associated with infection of the central nervous system. The review described recent data regarding epidemiology, diagnosis, and clinical manifestations upon infection by enteroviruses, arboviruses (flaviviruses and alphaviruses), herpesviruses (alpha- and beta-herpesviruses), and prions. The authors highlighted that those infections are often neglected, especially in low- and middle-income countries, although they represent important public health problems, with high rates of morbidity and mortality. Therefore, a deeper understanding of these infections should further improve the current surveillance strategies and help in detecting the emergence/re-emergence of neurotropic viruses.
